# Diagnostic value of plasma-derived exosomal miR-223 for epithelial ovarian cancer

**DOI:** 10.1186/s12905-024-02976-6

**Published:** 2024-03-02

**Authors:** Li Yang, Zhihong Yang, Zhihui Liu, Na Qi, Lili Tao

**Affiliations:** 1 Department of Obstetrics and Gynecology, Tangshan Workers’ Hospital, Tangshan, China; 2 Department of Basic Medicine, Tangshan Vocational and Technical College, 120 Xinhua West Road, Lubei District, Tangshan, Hebei Province 063000 China

**Keywords:** Exosomes, miR-223, Epithelial ovarian cancers, Diagnosis

## Abstract

**Objectives:**

To evaluate the diagnostic value of plasma exosomal miR-223 and its combination with CA125 for the diagnosis of early-stage epithelial ovarian cancer (EOC).

**Patients and methods:**

Exosomes derived from the plasma of 78 EOC patients, 40 patients with epithelial benign ovarian tumors, and 52 healthy participants were isolated using the ultracentrifugation method and identified by transmission electron microscopy (TEM) and western blot.

**Results:**

The expression of exosomal miR-223 was significantly upregulated in the plasma of EOC patients compared to that in healthy subjects and patients with benign diseases. The combination of exosomal miR-223 and CA125 from plasma had an equivalent area under the ROC curve (AUC) to CA125 alone for discriminating between EOC and non-EOC cases, including healthy subjects and benign ovarian tumors. However, the AUC value of the combination was 0.944 (95% CI: 0.899–0.990) for differentially diagnosing early-stage EOC from healthy subjects, slightly higher than that of CA125 alone (0.928, 95% CI: 0.875–0.981), with a sensitivity and specificity of 0.9784 and 0.885, respectively.

**Conclusion:**

Our data suggest that plasma exosomal miR-223 can be used as a complement to CA125 to increase the diagnostic power for differentiating early-stage EOC from healthy subjects.

**Supplementary Information:**

The online version contains supplementary material available at 10.1186/s12905-024-02976-6.

## Introduction

Ovarian cancer (OC) is a leading death cause for gynecological cancers, characterized by poor prognosis and high mortality rates. The most prevalent type of ovarian cancer is epithelial ovarian cancer (EOC), accounting for 90% of cases. EOC originates from the flat surface epithelial cells that cover the ovary, subserosal inclusion cysts, or the fimbriated end of the fallopian tubes [1]. Unfavorable outcomes in OC are mainly attributed to late-stage diagnosis, with an overall 5-year survival rate below 30% [2]. Conversely, early-stage boasts a 5-year survival rate exceeding 90%, highlighting the potential for improved prognoses through accurate early-stage diagnosis. Presently, serum cancer antigen 125 (CA125) test and transvaginal ultrasonography (TVUS) are commonly used screening tools for OC [3]. However, they often fail to detect OC at earlier stage, leading to limited reduction in OC-related mortality. Notably, only 50–60% of stage I-II OC patients exhibit increased CA125 levels [4]. Consequently, there is an urgent need for novel diagnostic markers to identify early-stage OC.

Exosomes are small membranous particles (40–160 nm), which are released into the extracellular space through the fusion of multivesicular bodies with the cell membrane [5]. They transmit functionally informative molecules, including proteins, nucleic acids, lipids, and even organelles into recipient cells to mediate intercellular communications [6]. Exosomes can be found in various body fluids, such as blood, urine, and cerebrospinal fluid. Accumulating evidence suggests that exosomes play fundamental roles in the progression, metastasis, and drug resistance of OC [7, 8]. MicroRNAs (miRNAs) are one of most abundant types in the RNA cargo of exosomes [9]. Exosomal miRNAs are usually tumor specific [10], thus attracting increasing attention in cancer diagnosis and prognosis due to their non-invasiveness, easy accessibility and stability [11]. For instance, plasma levels of exosomal miR-139-3p may serve as a novel biomarker for the early diagnosis and metastasis monitoring in colorectal cancer [12]. Plasma exosomes of breast cancer patients show selective enrichment of miR-1246, which is significantly increased compared to healthy controls [13].MiR-223, a multifunctional miRNA, plays a central role in innate immunity, including regulating immune cell differentiation and macrophage polarization, it also has immunomodulatory effects in certain tissues [14]. Recently, numerous studies have reported that miR-223 is involved in the development of varius types of cancer including hepatocellular [15], gastro-esophageal [16], breast [17] and lung cancers [18]. Furthermore, miR-223 has been considered a potential biomarker in several cancer types, including gastric cancer, non-small cell lung cancer (NSCLC), breast cancer, and recurrent ovarian cancer. Interestingly, circulating levels of miR-223 have been found to correlate with different stages of tumorigenesis and can be easily measured using molecular biology assays. For example, miR-223 is regarded as a reliable biomarker for early-stage NSCLC Furthermore, miR-223 has been considered as a possible biomarker in several types of cancers, including gastric cancer, non‐small cell lung cancer (NSCLC), breast cancer, and recurrent ovarian cancer. Interestingly, circulating levels of miR-223 have been found to correlate with different stages of tumorigenesis and can be easily measured using molecular biology assays. For example, miR-223 is regarded as a reliable biomarker for early-stage NSCLC [19], and low serum miR-223 levels are associated with poor outcome in patients with acute myeloid leukemia [20]. Increased levels of miR-223-3p level have been reported in ovarian cancer tissue and may act as a potential biomarker in recurrent ovarian cancer [21]. However, the use of plasma exosomal miR-223 as biomarkers in EOC has been rarely reported. In this study, we compared the expression levels of plasma exosomal miR-223 among 78 EOC patients, 40 patients with epithelial benign ovarian tumors, and 52 healthy controls, we also evaluated its diagnostic value by analyzing clinical characteristics in these patients.

## Materials and methods

### Patients and plasma samples

A total of 78 EOC patients who underwent tumor resection or primary debulking surgery at Tangshan Workers’ Hospital between March 2020 and October 2022 were enrolled in this retrospective study. The inclusion criteria were as follows: all patients were newly diagnosed with EOC at International Federation of Gynecology and Obstetrics (FIGO) stages I-IV and suitable for surgical treatment. Staging surgery was performed for suspected early-stage OC (Stage I or II) to determine the stage and remove the cancer. For advanced-stage OC (Stage III or IV), where the cancer has spread extensively, debulking or cytoreductive surgery was performed to remove all visible cancer [22]. All patients were confirmed to have EOC through postoperative pathological examination.

In addition, 40 age-matched patients with epithelial benign ovarian tumors (19 benign serous cystadenoma, 11 mucinous cystadenoma, 6 acinar cell tumors, and 4 mature teratomas) and 52 healthy controls (HC) were included in the study. EOC patients who had received anti-tumor treatment before surgery were excluded from the study. The healthy volunteers did not have any tumors or other immune and metabolic diseases. Written informed consent was obtained from all participants for the use of their blood samples. The present study was approved by the institutional research ethics committee of Tangshan Workers’ Hospital.

Blood samples (10 mL) were collected from each participant into a plasma separator tube and processed within an hour. Plasma was obtained by centrifugation at 2000×g for 10 min at 4 °C to remove living cells and cell debris. The supernatant was collected and stored at -80 °C for further analysis.

### Plasma exosomes isolation

Plasma exosomes were extracted using the ultracentrifugation method as previously described [23]. Briefly, the plasma was thawed at 4 °C and centrifuged at 3000×g for 10 min to remove debris. The supernatant was then ultracentrifuged at 10,000×g for 30 min at 4 °C using an ultracentrifuge (Beckman Coulter, Brea, CA, USA). Subsequently, another ultracentrifugation step was performed at 100,000×g for 120 min at 4 °C to enrich exosomes. The pellet was washed in 20 mL of phosphate-buffered saline (PBS) and collected by ultracentrifugation at 100,000×g for 120 min at 4 °C. After resuspension in PBS, the exosomes were stored at -80 °C for further analysis.

### Transmission electron microscopy (TEM)

Exosome pellets were thawed at 4 °C and resuspended in PBS. A 20 µL suspension was placed onto a mesh with a diameter of 2 nm carbon-coated copper grid. After draining with filter paper, the samples were fixed with 1% glutaraldehyde for 5 min. The grid was then washed five times with ddH2O and negatively stained with 3% phosphotungstic acid at room temperature for 3 min. After three additional washes with ddH2O for 15 min each, the grid was dried in air at room temperature for 5–10 min. The exosomes were observed and photographed under a transmission electron microscope (Thermo Fisher Scientific, Waltham, MA, USA).

### Western blotting analysis

A2780 cells and plasma-derived exosomes were lysed with radioimmunoprecipitation lysis buffer (Beyotime, Jiangsu, China), and total protein was obtained by centrifugation at 12,000×g for 15 min at 4 °C. After quantification using a bicinchoninic acid (BCA) Protein Assay Kit (Beyotime), equal amounts of cellular and exosomal protein were separated by SDS-PAGE and transferred to PVDF membranes (Millipore, Billerica, MA, USA). The membrane was blocked with 10% defatted milk in Tris-buffered saline containing 0.1% Tween 20 (TBST) for 1 h and then incubated overnight at 4 °C with primary antibodies, including anti-GM130 (Abcam ab52649; 1:1000), anti-HSP70 (Abcam ab2787; 1:1000), and anti-CD9 (Abcam ab236630; 1:1000). After washing with TBST three times, the membrane was incubated with appropriate HRP-conjugated secondary antibodies: goat anti-rabbit IgG H&L (HRP) (Abcam ab97051; 1:2000) or goat anti-mouse IgG H&L (HRP) (Abcam ab205719; 1:2000), as appropriate, for 1 h at room temperature. Finally, the protein bands were visualized on photographic films using the BeyoECL Plus chemiluminescence kit (Beyotime) with an electro-chemiluminescent system (PerkinElmer Life Science, Waltham, MA, USA).

### Exosomal miRNA extraction, reverse transcription and real-time quantitative polymerase chain reaction (RT-qPCR)

Total exosomal miRNA was extracted using the miRNeasy Micro kit (Qiagen, Hilden, Germany) following the manufacturer’s protocol. RNA quality and quantity were determined using the Agilent Bioanalyzer 2100 System (Agilent Technologies, Santa Clara, CA, USA) according to the manufacturer’s standard procedures. Quantification of exosomal miR-223 was assessed by RT-qPCR. Firstly, total exosomal miRNA was reverse-transcribed into cDNA using a miScript II RT kit (Qiagen) with Hispec buffer, following the manufacturer’s instructions. Briefly, mature miRNAs were polyadenylated by poly(A) polymerase and reverse transcribed into cDNA using oligo-dT primers. Polyadenylation and reverse transcription were performed in parallel in the same tube. The oligo-dT primers have a 3’ degenerate anchor and a universal tag sequence on the 5’ end, allowing amplification of mature miRNA in the real-time PCR step. Amplification of the cDNA was performed using target-specific miScript Primer Assays and the miScript SYBR Green PCR Kit, which contains the miScript Universal Primer (reverse primer) and QuantiTect SYBR Green PCR Master Mix (Qiagen), according to the manufacturer’s instructions. The target-specific primers were as follows: miR-223 forward, 5’-AGCCGTGTCAGTTTGTCAAAT-3’; reverse, 5’-GTGCAGGGTCCGAGGTC-3’; U6 forward, 5’-CTCGCTTCGGCAGCACA-3’, and reverse, 5’-AACGCTTCACGAATTTGCGT-3’. The 20-µL reaction system for the qPCR detection of miR-223 and U6 contained 10 µL 2x QuantiTect SYBR Green PCR Master Mix, 2 µL 10x miScript Universal Primer (reverse primer), 2 µL 10x miScript Primer Assay (specific forward primer), 4 µL dH2O, and 2 µL cDNA. The qPCR conditions were as follows: pre-denaturation at 95 °C for 10 min, followed by 40 cycles of denaturation at 95 °C for 15 s and annealing/extension at 60 °C for 60 s on a CFX-96 real-time PCR detection system (Bio-Rad). Small nuclear RNA U6 (U6 snRNA) was used as an internal reference, and the relative quantification was calculated using the 2-ΔΔCt method, where ΔCt = Ct [miRNA] - Ct [U6].

### Plasma CA125 quantification

Plasma CA125 levels were quantified using a commercially available ELISA kit (R&D Systems) following manufacturer’s instructions. Briefly, after adding 100 µL of Assay Diluent RD 1X to each well of a 96-well strip plate, 100 µL of standard, control, or 2-fold dilution EDTA plasma was added to each well, and the plate was incubated for 2 h at room temperature on a horizontal orbital microplate shaker. After washing each well for four times, 200 µL of Human CA125 Conjugate was added to each well and was incubated for 2 h at room temperature on the shaker. Then, 200 µL of Substrate Solution was added to each well and was incubate for 30 min at room temperature protecting from light. Finally, 50 µL of Stop Solution was added to each well. The optical density (OD) of each well was determined using a microplate reader set to 450 nm within 30 min. CA125 concentration was calculated according to the standard curve.

### Statistical analysis

Statistical analysis was performed using SPSS20.0 (IBM, Chicago, IL, USA) and GraphPad Prism 9 (GraphPad Software, San Diego, CA, USA). Data were presented as the mean ± standard deviation (SD), unless otherwise specified. One-way ANOVA was used to analyze comparisons among more than two groups. A *p*-value ≤ 0.05 was considered to be statistical significance. Spearman correlation analysis and linear regression were performed to test the correlation between plasma exosomal miR-223 and plasma CA125. Receiver operating characteristic (ROC) curve analysis and the area under the curve (AUC) were used to evaluate diagnostic value for plasma exosomal miR-223, CA125 and their combination. The cut-off values for exosomal miR-223 and CA125 in EOC diagnosis was determined using the Youden method, selecting the cut point at which Youden index (sensitivity + specificity − 1) is maximized.

## Results

### Identification of isolated plasma exosomes

**Fig. 1:**
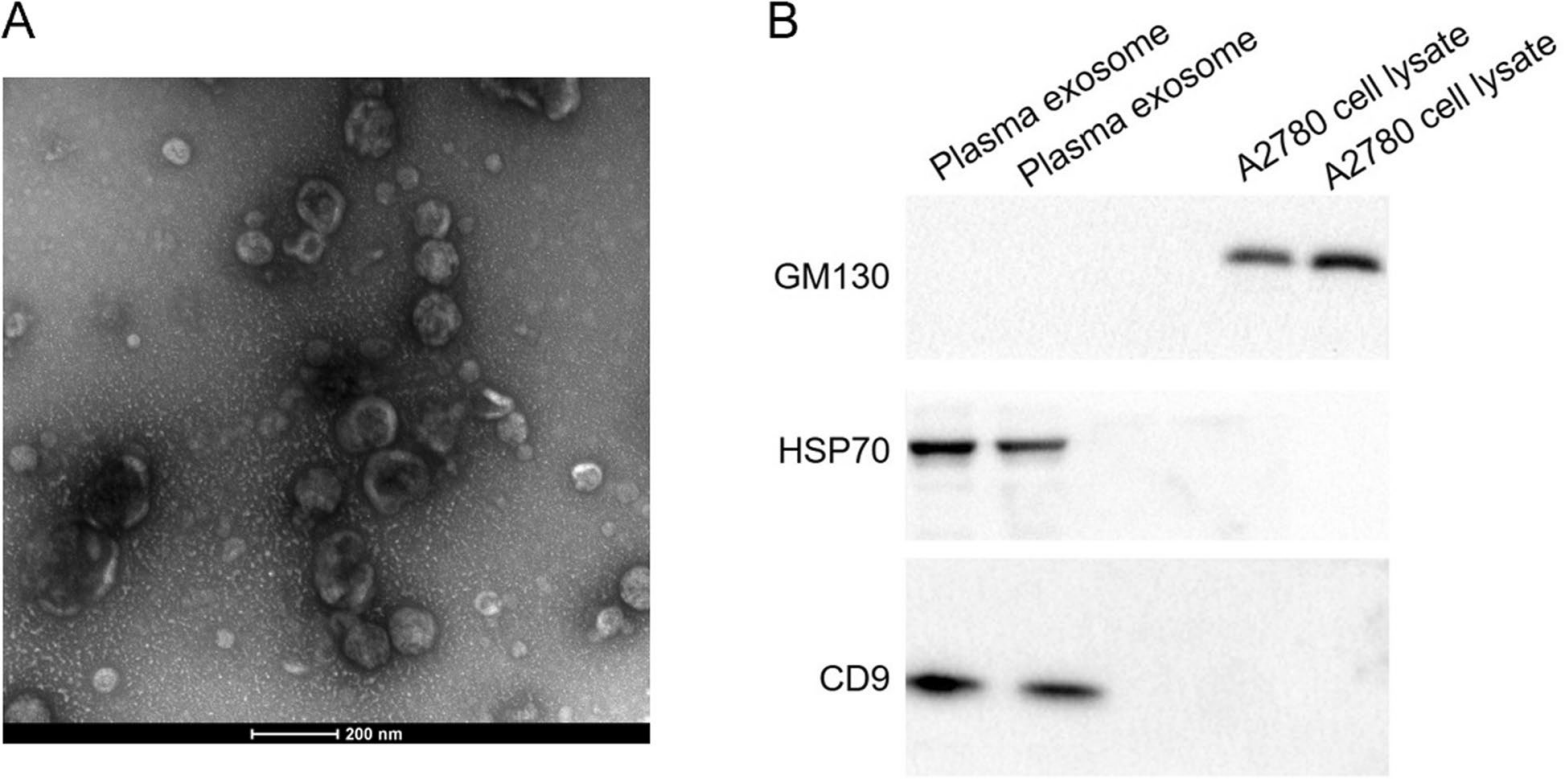
Verification of exosomes isolated from plasma. **a** Transmission electron microscopy (TEM) showed the size and morphology of the exosomes isolated from plasma. **b** Western blotting analysis of characteristic markers of extracellular vesicles, including HSP 70 and CD9, and cell marker GM130

We first verified whether exosomes were successfully extracted from plasma by ultracentrifugation. TEM revealed that the particles isolated from plasma were round or bowl-shaped macrovesicles with diameters ranging from 30 to 100 nm (Fig. 1A), exhibiting the typical appearance of exosomes. Western blot analysis showed that exosomes were positive for the exosomal markers HSP70 and CD9, and negative for the cis-Golgi marker GM130. In contrast, A2780 cell lysate was positive for GM130 and negative for HSP70 and CD9 (Fig. 1B). These results demonstrated the successful isolation of plasma exosomes.

### Plasma exosomal miR-223 expression at different clinical stages for EOC patients

**Fig. 2:**
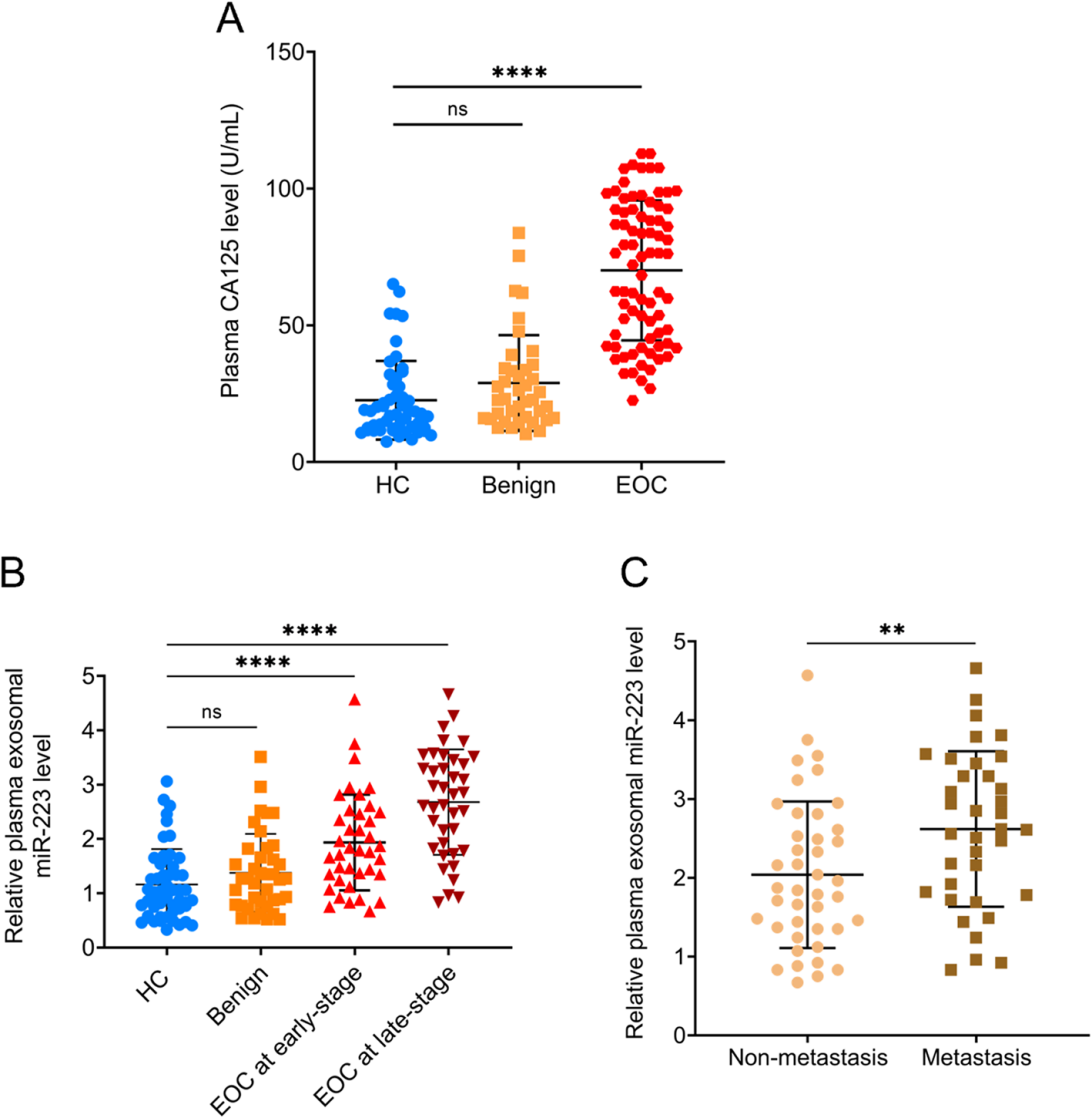
Plasma exosome-derived miR-223 levels at different EOC stages. **A** Plasma CA125 expression levels in plasma were compared among EOC patients, patients with benign disease and healthy subjects. **B **Plasma exosomal miR-223 levels compared among healthy subjects, patients with benign disease and EOC patients at different FIGO stage. **C** Exosomal miR-223 expression levels in plasma were significantly upregulated in metastatic EOC patients when compared with non-metastatic EOC patients. (**P < 0.01, ***P < 0.001, ****P < 0.0001, ns, not significant)

The clinical characteristics of all 78 EOC patients are shown in Table 1. We examined plasma exosomal miR-223 and CA125 levels in EOC patients (EOC group), patients with epithelial benign ovarian tumors (benign group), and healthy controls (HC group) using RT-qPCR and ELISA, respectively. As shown in Fig. 2A, similar to plasma CA125 levels, exosomal miR-223 levels in EOC patient plasma were significantly increased (*P* < 0.0001) compared to those in benign patients and healthy controls. We also investigated plasma exosomal miR-223 levels in EOC patients at different FIGO stages. As shown in Fig. 2B, exosomal miR-223 expression levels in patients at FIGO stage III + IV were markedly higher (*P* < 0.001) than those in patients at stage I + II. Moreover, exosomal miR-223 expression levels in metastatic EOC patients were significantly higher (*P* < 0.01) than non-metastatic EOC patients (Fig. 2C).

**Table 1:** Clinical characteristic of the EOC patients

Total number	78
Age (years)	
Median (min-max)	57 (31, 79)
**Menopause**	
Yes	51
No	27
**Pathological type**	
Serous	54
Clear cell	10
Mucinous	7
Endometrioid	4
Others	3
**FIGO stage**	
I	30
II	9
III	25
IV	14
**Metastasis (lymph nodes and distant)**	
Yes	36
No	42
**CA125 (U/mL)**	
Median (quartile intervals)	75.65 (44.75, 92.45)

### Diagnostic value of plasma exosomal miR-223 for epithelial ovarian cancer

**Fig. 3:**
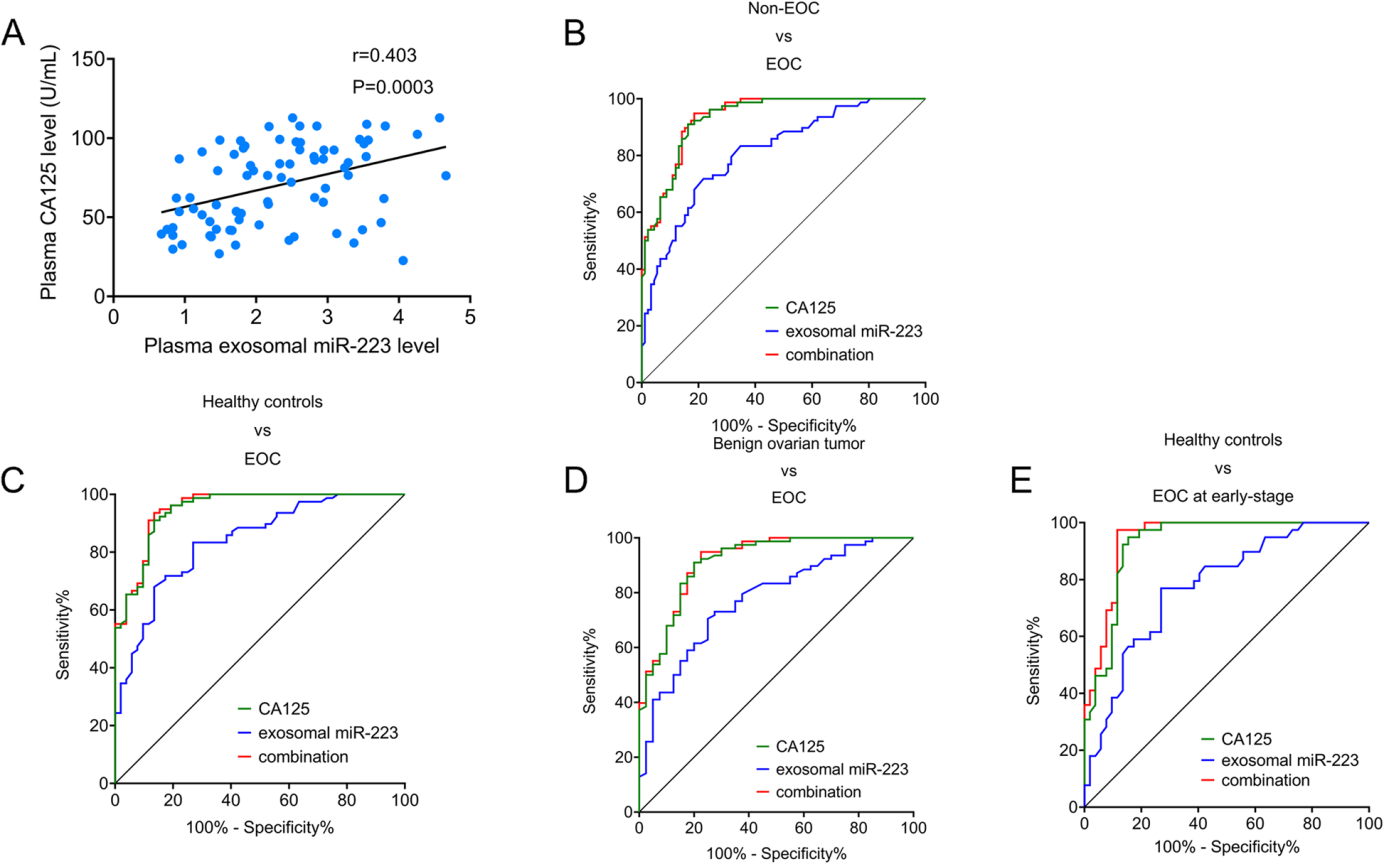
Diagnostic power of plasma exosomal miR-223 for EOC. **a** Correlation analysis of plasma exosomal miR-223 with CA125 in EOC group. **b** ROC curve analysis for exosomal miR-223, CA125, and combined indicators for EOC patients relative to non-EOC subjects. **c** ROC curve analysis for exosomal miR-223, CA125, and combined indicators for EOC patients relative to healthy subjects. **d** ROC curve analysis for exosomal miR-223, CA125, and combined indicators for metastatic EOC patients relative to non-metastatic EOC patients. **e** ROC curve analysis for exosomal miR-223, CA125, and their combination for discriminating EOC patients at early-stage from healthy subjects

We investigated potential correlations between plasma exosomal miR-223 levels and plasma CA125. As shown in Fig. 3A, exosomal miR-223 showed a positive correlation with plasma CA125 (*r* = 0.403, [CI: 0.1982 to 0.5740], *p* = 0.0003). The diagnostic value of exosomal miR-223 was evaluated using ROC curve analysis. A ROC curve was calculated for distinguishing EOC from non-EOC, comparing it with CA125. As shown in Table 2; Fig. 3B, the AUC (95% CI) for exosomal miR-223 and CA125 were 0.812 (95% CI: 0.749–0.876) and 0.933 (95% CI: 0.899–0.968), respectively. The AUC for their combination was 0.937 (95% CI: 0.903–0.970), which was equal to CA125 alone. Table 3; Fig. 3C showed that the AUC (95% CI) for exosomal miR-223 and CA125 in distinguishing EOC from healthy subjects was 0.839 (95% CI: 0.771–0.907) and 0.948 (95% CI: 0.912–0.984), respectively. Their combination did not improve the diagnostic value, with an AUC of 0.95 (95% CI: 0.903–0.970) compared to CA125 alone. Table 4; Fig. 3D displayed the AUC (95% CI) for exosomal miR-223 and CA125 as 0.778 (95% CI: 0.692–0.864) and 0.914 (95% CI: 0.859–0.969), respectively, when discriminating EOC from a benign ovarian tumor. Their combination did not add any diagnostic value to CA125 alone, with an AUC of 0.918 (95% CI: 0.864–0.971). Furthermore, we evaluated the diagnostic value of exosomal miR-223 and CA125 in discriminating early-stage EOC from healthy subjects. The results in Fig. 3E; Table 5 showed that the AUC value of the combination was 0.944 (95% CI: 0.899–0.990), which was slightly higher than that of CA125 alone (AUC = 0.928, 95% CI: 0.875–0.981), with a sensitivity and specificity of 0.9784 and 0.885, respectively.

**Table 2:** Diagnostic value of plasma exosomal miR-223 and CA125 for EOC patients relative to non-EOC subjects

	cutoff value	AUC (95% CI)	sensitivity	specificity	Youden index
Plasma CA125	37.2 U/mL	0.933 (0.899–0.968)	0.91	0.837	0.747
Plasma exosomal miR-223	1.655	0.812 (0.749–0.876)	0.718	0.783	0.501
Combination	-	0.937 (0.903–0.970)	0.949	0.815	0.764

**Table 3:** Diagnostic value of plasma exosomal miR-223 and CA125 for EOC patients relative to healthy subjects

	cutoff value	AUC (95% CI)	sensitivity	specificity	Youden index
Plasma CA125	37.2 U/mL	0.948 (0.9120.984)	91.0%	86.5%	0.776
Plasma exosomal miR-223	1.345	0.839 (0.771–0.907)	83.3%	73.1%	0.564
Combination	-	0.952 (0.918–0.987)	93.6%	86.5%	0.801

**Table 4:** Diagnostic value of plasma exosomal miR-223 and CA125 for EOC patients relative to benign diseases patients

	cutoff value	AUC (95% CI)	sensitivity	specificity	Youden index
Plasma CA125	36.6 U/mL	0.914 (0.859–0.969)	91.0%	80.0%	0.710
Plasma exosomal miR-223	1.625	0.778 (0.692–0.864)	73.1%	72.5%	0.456
Combination	-	0.918 (0.864–0.971)	94.9%	77.5%	0.724

**Table 5:** Diagnostic value of plasma exosomal miR-223 and CA125 for EOC patients at early-stage relative to healthy controls

	cutoff value	AUC (95% CI)	sensitivity	specificity	Youden index
Plasma CA125	34.85 U/mL	0.928 (0.875–0.981)	94.9%	84.6%	0.795
Plasma exosomal miR-223	1.345	0.778 (0.683–0.873)	76.9%	73.1%	0.500
Combination	-	0.944 (0.899–0.990)	97.4%	88.5%	0.859

## Discussion

In clinical practice, the early diagnosis and prognosis assessment of cancer pose significant challenges and have garnered considerable attention. Exosomes, in recent years, have been identified as playing crucial roles in cancer progression by transferring biomolecules such as proteins, mRNA, and miRNA between cells [15]. Recent studies have suggested that circulating exosomal miRNAs could serve as potential biomarkers for cancer diagnosis and prognosis due to their stability and non-invasive sampling methods [16]. Notably, serum exosomal miR-378 has been reported as a promising non-invasive biomarker for screening and monitoring non-small-cell lung cancer [24], while serum exosomal miR-1910-3p combined with CA153 has shown improved sensitivity for breast cancer diagnosis [25]. In addition, miR-106b-3p derived from serum exosomes has been proposed as a potential prognostic biomarker for distinguishing metastatic colorectal cancer (mCRC) patients from CRC patients without metastasis [26].

In this study, we investigated plasma exosomal miR-223 levels in EOC patients and compared them with benign disease patients and healthy subjects. Our findings revealed up-regulation of plasma exosomal miR-223 levels in EOC patients, distinctly differentiating them from the healthy control group. These results align with a previous study by Fang et al [27], which demonstrated significantly higher miR-223 levels in ovarian cancer tissues compared to adjacent non-cancerous tissues. Furthermore, aberrant expression of miR-223 was significantly correlated with histological grade, lymph node metastasis, and FIGO stage. Additionally, we observed significantly elevated exosomal miR-223 levels in metastatic EOC patients compared to non-metastatic patients. Moreover, when comparing EOC patients at FIGO stages I + II to those at stages III + IV, exosomal miR-223 levels were significantly increased. Thus, our findings suggest a positive correlation between plasma exosomal miR-223 levels and EOC disease progression, serving as a potential reflection of disease severity. Notably, elevated miR-223 expression has also been observed in other cancers, including CRC [28], gastric cancer [29], and hepatocellular carcinoma (HCC) [30]. These results indicate the possible role of miR-223 as an oncogene involved in multiple cancers.

Finally, we evaluated the diagnostic power of exosomal miR-223, CA125, and their combinations. The combination of these markers exhibited an AUC of 0.937 for discriminating EOC from non-EOC, with 94.9% sensitivity and 81.5% specificity. Moreover, the combination achieved an AUC of 0.952 for discriminating EOC from healthy controls, with 93.6% sensitivity and 86.5% specificity, which was equivalent to using CA125 alone. Interestingly, the combination showed an AUC value of 0.944 (95% CI: 0.899–0.990), slightly higher than that of CA125 alone (AUC = 0.928, 95% CI: 0.875–0.981), demonstrating relatively higher sensitivity and specificity at 0.9784 and 0.885, respectively. These results suggest that exosomal miR-223 may serve as a novel complementary marker to CA125 for the early diagnosis of EOC. It is worth noting that the data for EOC at early-stage vs. late-stage (Fig. 2B) and non-metastasis vs. metastasis (Fig. 2C) appear similar but not identical. This similarity may arise from the fact that EOC patients at early stages were all non-metastatic, while patients with metastasis were all at late stages, resulting in overlapping patients between the early-stage and non-metastasis groups, as well as the late-stage and metastasis groups.

There were several limitations in the present study. Firstly, the long-term prognostic efficacy of exosomal miR-223 was not investigated due to a lack of clinical follow-up data for each EOC patient. Secondly, due to the small sample size, further research with a larger cohort is required to confirm the findings of the current study. Thirdly, we did not investigate the mechanism of abnormal exosomal miR-223 expression in EOC, which warrants future investigation.

## Conclusion

Plasma exosomal miR-223 was significantly up-regulated in EOC patients and correlated with disease progression. Our study clearly demonstrates that exosomal miR-223, as a non-invasive biomarker, may complement CA125 to enhance the diagnostic accuracy for differentiating early-stage EOC from healthy subjects.

### Supplementary Information

**Supplementary Material 1.**

## Data Availability

The data used and/or analyzed during the current study are available from the corresponding author.
