# Supplementary material for: Diagnostic value of plasma-derived exosomal miR-223 for epithelial ovarian cancer
Source: BMC Womens Health. 2024 Mar 2;24:150. doi: 10.1186/s12905-024-02976-6 (PMC10908149; doi:10.1186/s12905-024-02976-6)
Supplement: Supplementary file 1 — Supplementary Material 1. [file 12905_2024_2976_MOESM1_ESM.pdf]

FIGURE 1B

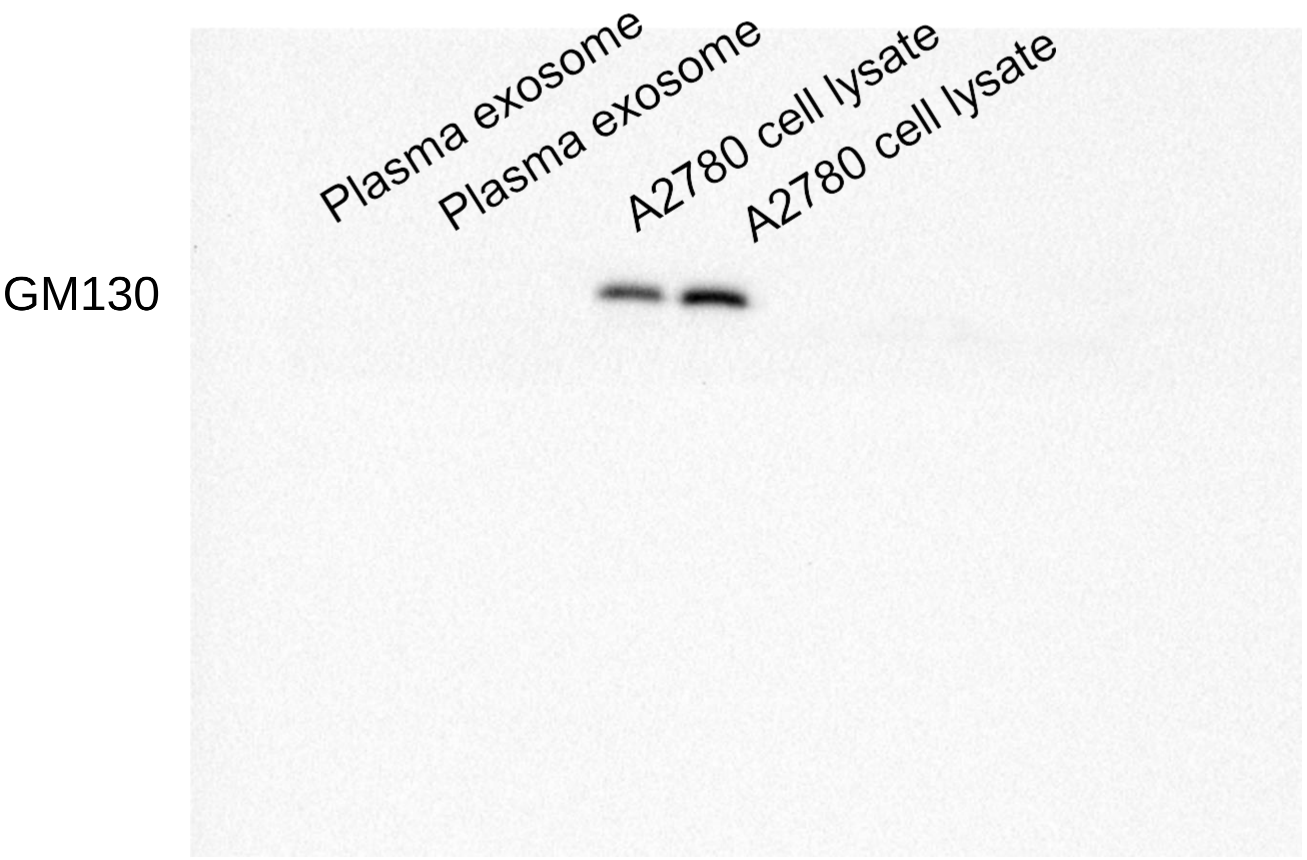

This is the original western blot image of GM130, which is the cell marker of Figure 1B.

FIGURE 1B

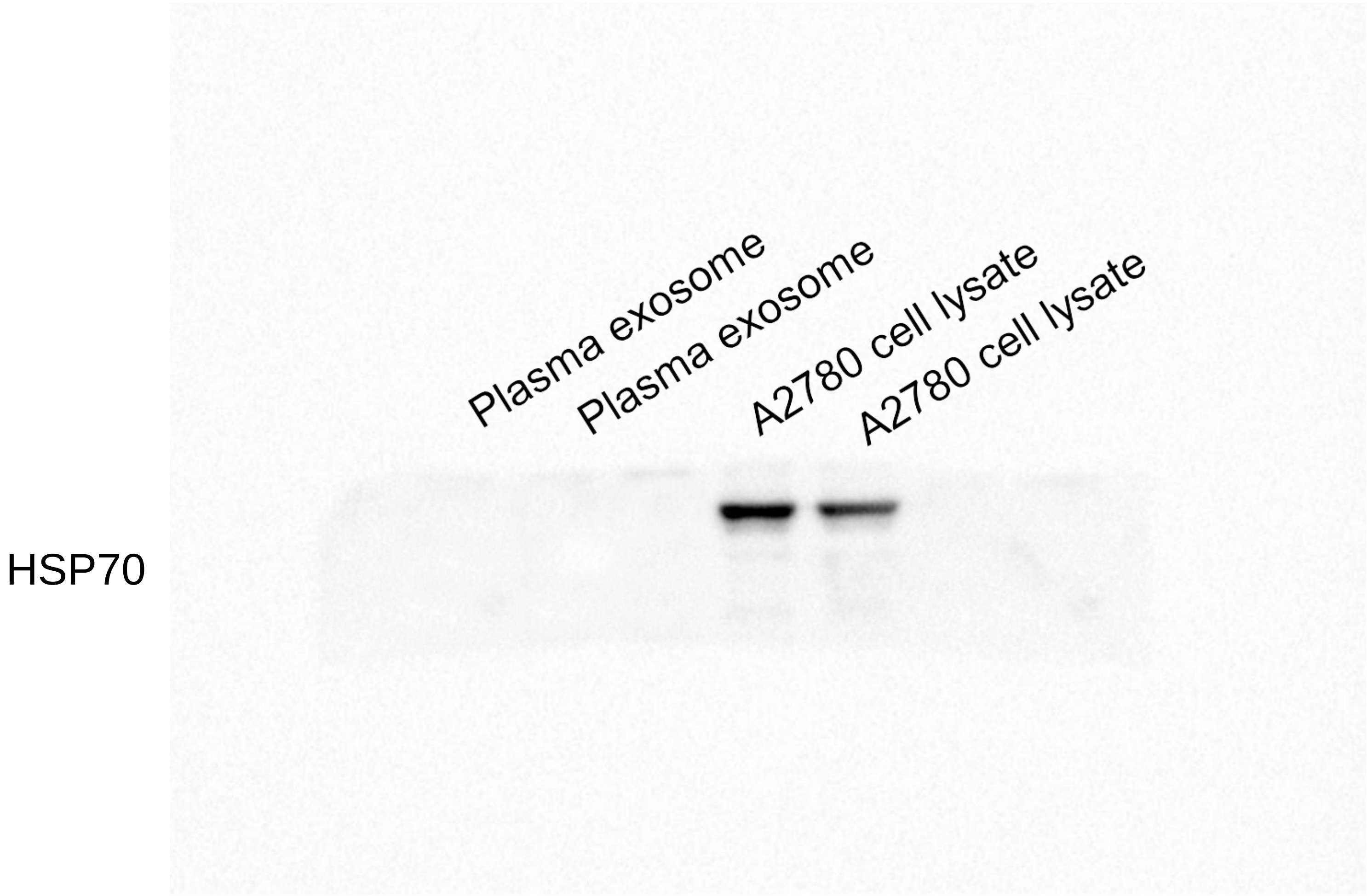

This is the original western blot image of HSP70, which is the characteristic marker of Figure 1B.

FIGURE 1B

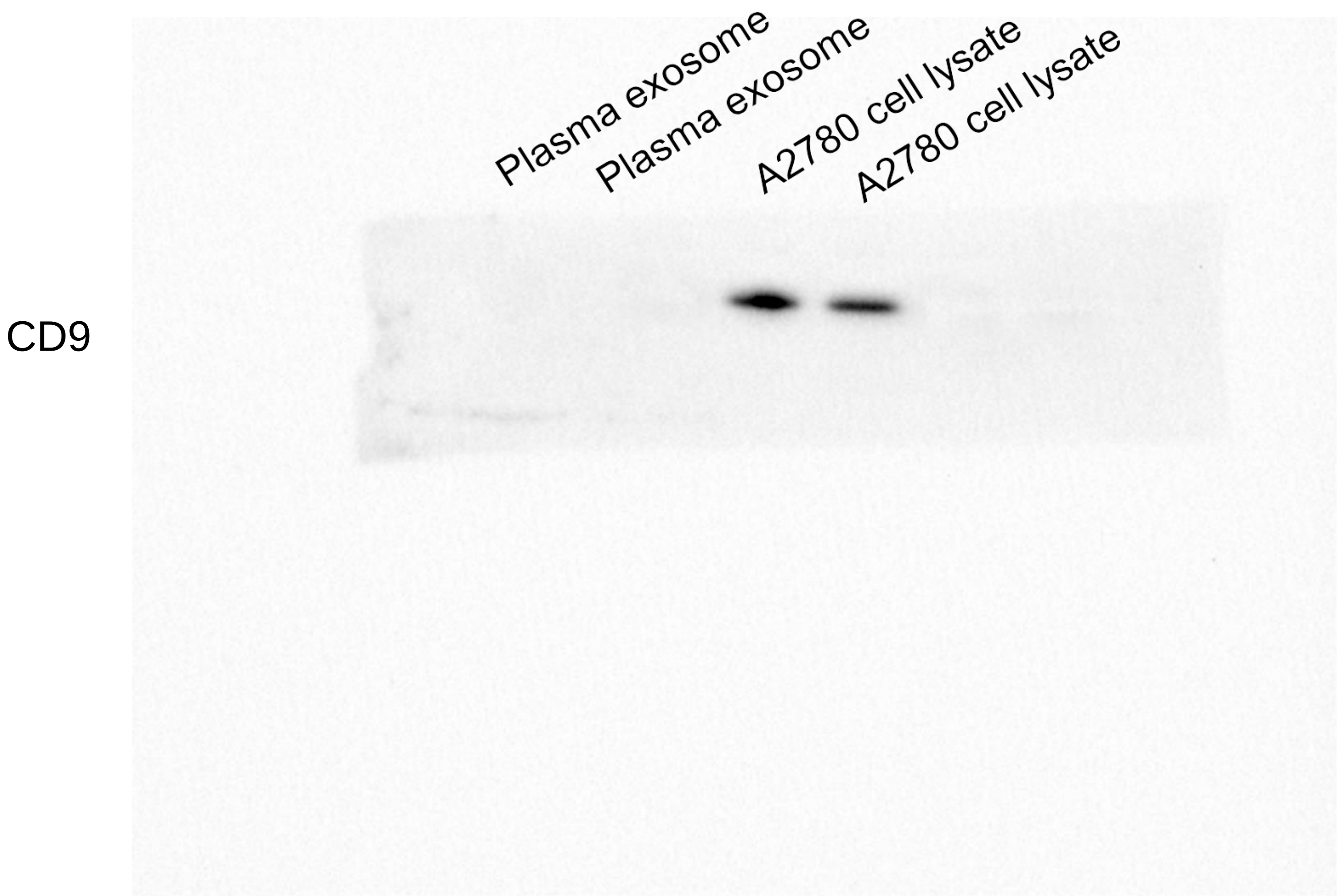

This is the original western blot image of CD9, which is the characteristic marker of Figure 1B.

Were the blots cut prior to hybridisation with antibodies? Yes.

The marker was only cut off at the membrane transfer step to determine its molecular weight. The first antibody cannot bind, and the marker cannot be seen when the second antibody is exposed after incubation.

Western blotting of characteristic markers of extracellular vesicles, including HSP 70 and CD9, and cell marker GM130.

This experiment is a validation experiment and has not been statistically analyzed, without conducting three repeated experiments.
